# Interferon beta-1a for COVID-19: critical importance of the administration route

**DOI:** 10.1186/s13054-020-03048-5

**Published:** 2020-06-12

**Authors:** Juho Jalkanen, Maija Hollmén, Sirpa Jalkanen

**Affiliations:** 1 Faron Pharmaceuticals Ltd., Turku, Finland; 2 MediCity Research Laboratory and Institute of Biomedicine, University of Turku, Turku, Finland

Type I interferons, especially IFN-beta, have been appointed as potential leading therapeutics to tackle severe COVID-19 and are currently being evaluated in REMAP-CAP and the WHO’s Solidarity Trial. As a most recent example, combination treatments with IFN-beta, lopinavir-ritonavir, and ribavirin showed that the arm containing IFN-beta was superior in eliminating the virus from the nasopharyngeal swabs in phase II clinical trial [1]. Recent papers on the matter unfortunately fall short of differentiating between subcutaneous (s.c.) and intravenous (i.v.) administration, which are completely different treatments concerning drug exposure and wanted effects in the lung endothelium, which is under attack in COVID-19 [2]. We wish to highlight the differences of these two treatment methods and also other crucial aspects of IFN-beta treatment for COVID-19 and acute respiratory distress syndrome (ARDS).

A recent report concluded that the pharmacological effects of s.c. vs. i.v. IFN-beta-1a are the same, because they produce similar anti-viral responses [3]. Importantly, however, the pharmacokinetics of s.c. vs. i.v. IFN-beta are complete mirror images, “flip-flops” [4]. Maximum serum concentrations (Cmax) and total exposure through serum concentrations are significantly higher after i.v. than s.c. injections (*p* = 0.0001). Prior corner stone PK studies investigating s.c. vs. i.v. administration of IFN-beta-1a conclude that s.c. administration produces significantly lower drug concentrations and incomplete bioavailability compared to i.v. dosing. The bioavailability via the s.c. route is about one third of that obtained by i.v. injections [4]. For critically ill patients on vasopressors and with very limited peripheral microcirculation, the bioavailability of s.c. dosed IFN-beta becomes even more questionable. IFN-beta is cleared almost solely through its receptor (IFNAR). With s.c. dosing, IFN-beta is slowly taken up by the lymphatic system, from which it enters the blood during a number of hours with modest peak concentrations. In contrast, i.v. dosing achieves high serum concentration and efficiently reaches vast capillary beds of central organs, where it is taken up by its receptors without saturating the body and causing unwanted adverse events. This is an important aspect as endothelial dysfunction is connected to COVID-19 infection [2, 5]. Nonetheless, the purpose of i.v. administered IFN-beta for the treatment of COVID-19 and ARDS is to maximise bioavailability of the drug at the lung vasculature, as well as other vascular beds. This is hardly achieved with s.c. dosing in critically ill patients. IFN-beta increases CD73 in pulmonary capillaries. This is of utmost importance as CD73 is the key enzyme for vascular integrity under hypoxic conditions. The protective effect of IFN-beta on the lung is attributed to the clearance of pro-inflammatory ATP and pro-thrombotic ADP from circulation and converting them into highly anti-inflammatory adenosine via AMP step by CD73 [6].

It is well known that corticosteroids as immunosuppressors dampen our natural anti-viral responses, and the direct inhibitory effect of corticosteroids on IFN signalling has been reported [7]. Still corticosteroids are widely used to treat ARDS and severe viral respiratory infections even though several studies have shown that corticosteroid use is associated with harm in viral outbreaks such as H1N1 and MERS [8]. In fact, reports on using type I IFNs for the treatment of MERS reveal that the majority of these patients received systemic corticosteroids with IFN. For example, 60% of MERS patients received corticosteroids with type I IFN [9]. In the recent INTEREST study investigating the use of i.v. IFN-beta-1a for ARDS, the primary analyses did not show any benefit for IFN-beta over placebo [10]. However, nearly 60% of the patients received corticosteroids with IFN-beta. Further studies revealed that corticosteroids block IFN signalling and the upregulation of CD73 expression in human pulmonary endothelial cells, and combining i.v. IFN-beta with systemic corticosteroids may be even more detrimental than corticosteroids alone [11]. These findings suggest that the different anti-inflammatory pathways triggered by IFN-beta and corticosteroids should not be induced at the same time.

Immunomodulation is complex, and timing of the treatments is critical. There are a limited number of direct studies on the timing of immunomodulatory treatments such as IFN-beta, but given our basic understanding of human biology and viral defence, we suggest that IFN-beta should be given early to COVID-19 patients. In mild cases such as in the recent clinical trial, even s.c. administered IFN-beta was effective [1], but in more severe cases, i.v. injections are needed to rapidly reach the endothelium. As ARDS rises together with a cytokine storm, corticosteroids may play a beneficial role during the later fibrotic phase or just by calming down the cytokine storm after IFNs have had their impact. This is supported by Villar et al. who showed that the use of dexamethasone was associated with better survival in ARDS [12]. A notable feature of this study is that the enrolled patients were not on steroids when entering the trial. Thus, initial endogenous IFN responses had not been tampered. Sequential treatment strategy may be the future once we are able to reliably understand the time course of patients’ immunological responses.

Severely ill COVID-19 patients with increased levels of plasma cytokines (especially IL-6) show signs of immune exhaustion and poor IFN responses [13]. Even in such cases, these patients would most likely benefit from IFN-beta, because it is the most potent anti-viral and anti-inflammatory agent of all interferons. It can induce the desired immune boost, but simultaneously downregulate IL-6 and IL-8 [14] and impair extravasation of neutrophils into lungs [15].

## Conclusions

IFN-beta is now among the leading candidates to treat COVID-19 in various clinical trials, and i.v. and s.c. routes of administration are considered to be equal. This is not the case due to the different bioavailabilities of IFN-beta via i.v. and s.c. injections in target organs. This aspect needs to be taken seriously, when critically ill patients with compromised peripheral circulation are treated.

## Data Availability

NA
